# Compact Feeding Network for Array Radiations of Spoof Surface Plasmon Polaritons

**DOI:** 10.1038/srep22692

**Published:** 2016-03-07

**Authors:** Jun Jun Xu, Jia Yuan Yin, Hao Chi Zhang, Tie Jun Cui

**Affiliations:** 1 State Key Laboratory of Millimeter Waves, School of Information Science and Engineering, Southeast University, Nanjing 210096, China; 2 Synergetic Innovation Center of Wireless Communication Technology, Southeast University, Nanjing, 210096, China; 3 Cooperative Innovation Center of Terahertz Science, University of Electronic Science and Technology, Chengdu, 611731, China

## Abstract

We propose a splitter feeding network for array radiations of spoof surface plasmon polaritons (SPPs), which are guided by ultrathin corrugated metallic strips. Based on the coupled mode theory, SPP fields along a single waveguide in a certain frequency range can be readily coupled into two adjacent branch waveguides with the same propagation constants. We propose to load U-shaped particles anti-symmetrically at the ends of such two branch waveguides, showing a high integration degree of the feeding network. By controlling linear phase modulations produced by the U-shaped particle chain, we demonstrate theoretically and experimentally that the SPP fields based on bound modes can be efficiently radiated to far fields in broadside direction. The proposed method shows that the symmetry of electromagnetic field modes can be exploited to the SPP transmission network, providing potential solutions to compact power dividers and combiners for microwave and optical devices and systems.

Surface plasmon polaritons (SPPs) are two-dimensional electromagnetic (EM) fields, which are highly localized, propagate along metal-dielectric interfaces, and decay exponentially into adjacent media. Owing to the advantages of confined EM fields in subwavelength scales, SPPs have a lot of potential applications in super-resolution imaging, miniaturized sensors, and biosensing^1^,^2^,^3^,^4^. One of the major obstacles for the development of the SPP technology is the efficient manipulations of SPP fields^5^. Previous researches have been primarily focused on the excitation of SPP waveguide modes, and various types of SPP-based waveguide components have been suggested and demonstrated. Most of these functions rely on the coupling process of dipole antenna or plane-wave incidence in the optical frequency^6^,^7^,^8^, but the conversion has relatively low efficiency. In addition, the excitation of SPPs requires a particular oblique angle of incidence to achieve the wave vector required by SPPs. But minor deviations from such fixed incidence angle will lead to a significant reduction of efficiency, which greatly limits the practical applications of SPPs^9^.

Recently, in the microwave regime, an ultrathin corrugated metallic strip fed by a coplanar waveguide (CPW) has been investigated^10^,^11^, which converts the guided waves to spoof SPPs in broadband with high efficiency. Such a plasmonic waveguide provides the required localization of SPP fields, solving the problem that SPPs cannot be excited efficiently on flat metallic surfaces in the microwave and terahertz frequencies. Based on the ultrathin corrugated metallic structure, many new devices have been proposed, including SPP filters^12^,^13^, SPP frequency-selective devices^14^, switches^15^, and active SPP amplifier^16^.

Power splitter is an essential component for integrated circuit and system, which is also an important part of antenna array. Several concepts achieving the split of guided plasmons have been studied, e.g., based on symmetrical gratings on both sides of nanostructures^17^,^18^, Y-shaped splitters^19^,^20^, T-shaped splitters^21^,^22^,^23^, and splitters related to the multimode interference^24^,^25^,^26^,^27^. However, two problems need to be solved in the above-mentioned power splitters: how to guarantee the transmission of generated SPPs in the desired branch, and how to split the power according to a desired proportion.

On the other hand, the conversion of highly localized surface modes into spatial propagating waves is also very important, which could play a critical role in the plasmonic waveguide information links. A commonly used approach to achieve this goal is employing periodic subwavelength grating. The gradient-index metasurfaces have attracted considerable attention as the components of efficient energy coupling between SPP waves and spatial propagating waves^28^,^29^,^30^,^31^,^32^, in which the manipulation of reflection phases is fundamental for generating the synthetic scattering diagram of macroscopic object. The concept of gradient-index metasurface was also extended to leaky modes, offering new potentials for radiation applications^33^. By providing a means of controlling both amplitude and phase distributions across the linearly gradient index chains, the approach offers a pathway to construct directive emitting devices with good radiation properties that are similar to the conventional antenna arrays. By using the phase discontinuities, the wavefronts can be reshaped and a series of optical phenomena have been verified theoretically and experimentally^31^,^32^.

In this work, we propose a splitter feeding network for array radiations of spoof SPPs, in which efficient transition of SPP modes from a single corrugated metallic strip to two adjacently-parallel corrugated metallic strips. By adding phase-gradient linear U-shaped particle chains in the arms of two branch corrugated waveguides, the SPP waves can be effectively converted to far fields in the broadside direction. Here, the function of the U-shaped array is similar to the SPP antenna. The measurement results of S scattering parameters and near fields show excellent performance in the certain frequency band. Different from the traditional splitter feeding network, the proposed structure would modulate the SPP-wave propagation, resulting in variation of the SPP field distribution. Attributing to above feature, the proposed device is expected to have potential applications in highly integrated photonic circuits and phase-array SPP antennas.

## Result

**Figure 1:**
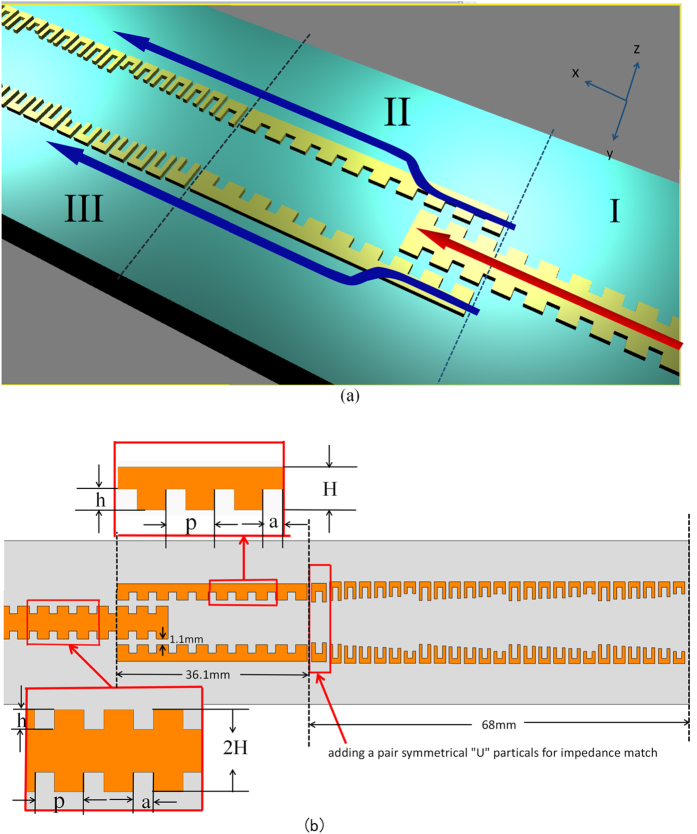
(**a**) Schematic view of the proposed splitter feeding network for array radiations of Spoof SPPs, which contains three regions (I, II and III). (**b**) The detailed configurations of Regions I and II, in which *p* = 3.76 *mm, a *= 1.5 *mm, h* = 1.8 *mm*, and *H *= 3.15 *mm*.

**Figure 2:**
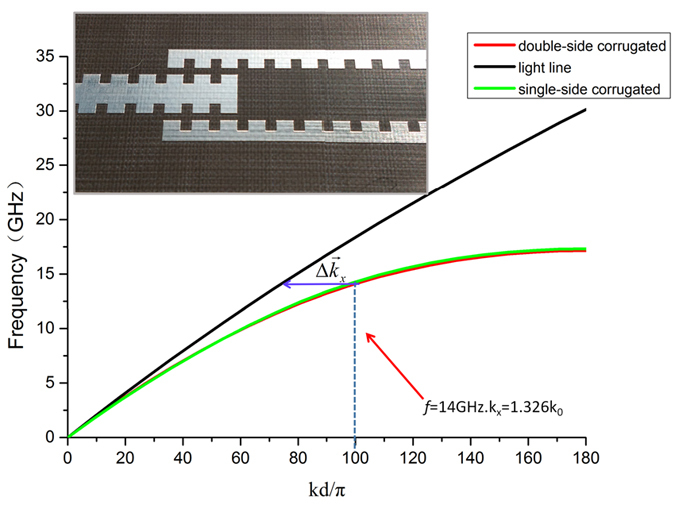
The dispersion relations of plasmonic waveguides with the double- and single-side corrugated metallic structures (see the inset for fabricated sample). The black line denotes the light line.

**Figure 3:**
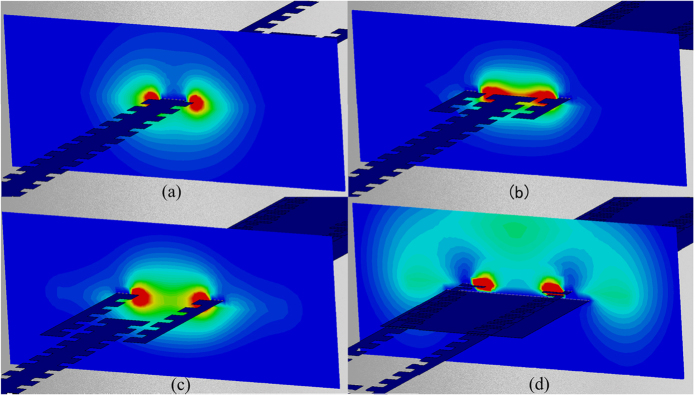
Simulated near-electric-field distributions (amplitudes) of the SPP feeding and radiation system at 14 GHz on four different observation planes perpendicular to the SPP propagation direction. (**a**) At the double-side corrugated metallic strip. (**b**) At the transition section of double- and single-side corrugated strips. (**c**) At the parallel single-side corrugated strips. (**d**) At the U-shaped particle array. In (**a**–**c**), it can be seen that most of EM fields are localized around the structure. It is clear that the EM fields are coupled from the double-side corrugated strip to the two oppositely oriented single-side corrugated strips with equal amplitude, which is similar to a 3-dB coupler. In (**d**), it can be seen that most of EM fields spread out the particle cells.

A three-dimensional (3D) view of the proposed structure is depicted in Fig. 1(a), which consists of three regions. The first region (Region I) is a double-side corrugated metallic strip, supporting spoof SPP propagations with the wavenumber *k*_*spp*_. This corrugated strip is connected to a 50 Ω coplanar waveguide (CPW) transmission line (not shown in Fig. 1(a)). Good matching of both momentum and impedance between CPW and the plasmonic waveguide ensures the high-efficiency conversion from spatial modes in CPW to SPP modes and high-efficiency transmission of SPP waves. In the double-side corrugated metallic strip, the period (*d*) and depth (*h*) of grooves have major influence on the cutoff frequencies of the dispersion relations, whereas the groove width (*a*) mainly improves the confinement ability of SPP fields. Therefore, we first choose the cutoff frequency, such as 16 GHz. Then the structure parameters are designed as: the depth of groove *h* = 1.8 *mm*, the substrate thickness *d* = 1 *mm*, the dielectric constant of the substrate *ε*_*r*_ = 3.5, the groove period *p* = 3.76 *mm*, and the width of grooves *a* = 1.5 *mm.* Figure 2 illustrates the dispersion relation of spoof SPPs on the corrugated metallic strip (red line). The amplitude of the near electric field (*E*_*x*_ component) was computed on a plane perpendicular to the double-side corrugated strip, as illustrated in Fig. 3(a). We clearly observe that the electric field is concentrated in both sides of grooves, which exhibits typical SPP properties.

The second region (Region II) is a transition of the double-side corrugated strip and two adjacently parallel single-side corrugated metallic strips. As presented in Fig. 1(b), two coupled single-side corrugated strips have opposite corrugation orientations. According to the coupled mode theory, the EM energy can be fully switched from one waveguide to another with the same propagation constants. By designing the geometrical parameters, spoof SPPs which have the similar dispersion relation and mode characteristics to the single double-side corrugated strip can be supported by the branch single-side corrugated strips. This can be clearly seen in Fig. 2, from which we note that the dispersion curves of two kinds of plasmonic waveguides (green and red lines) are nearly the same. The cutoff frequency of the double-side strip is slightly lower than that of single-side strip, both of which are around 14 GHz. From Fig. 2, we also observe that these dispersion curves significantly deviate from the light line, implying strong confinement of SPP modes for such two types of plasmonic waveguides.

Besides the similar dispersion relations, the consistency of electric-field modes supported by the two plamsonic waveguides has the same importance to decrease the reflections at the transition section. Here we compare the EM field distributions of the two kinds of plasmonic waveguides on planes perpendicular to the SPP propagation directions. As discussed earlier in Fig. 3(a), the double-side corrugated structure in fact supports a symmetric mode (even mode) of SPPs. The even SPP mode can then be coupled in the two adjacent single-side corrugated strips. Figure 3(b,c) demonstrate the electric field distributions in two different places in Region II, one of which is located at the connection part of the single double-side corrugated strip and two adjacent single-side corrugated strips (Fig. 3(b)), while the other of which is located at the two single-side corrugated strips (Fig. 3(c)). Comparing Fig. 3(a–c), we notice that the electric fields in the double-side corrugated strip can be regarded as the combination of two single-side corrugated strips. The electric-field distributions imply that the SPP fields are not distorted when they are coupled from the double-side corrugated strip into the two single-side corrugated branches, resulting in very low scattering at the node of transition.

**Figure 4:**
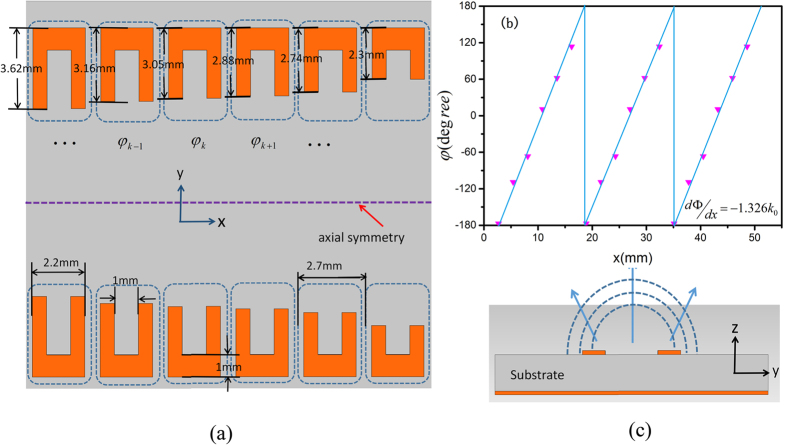
(**a**) A periodic unit of the SPP radiation array with the phase gradient designed as 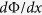 = 1.326 k_0_, which contains six U-shaped particles with different arm lengths. (**b**) Reflection phase profile for the gradient-phase particle array. (**c**) A schematic cross section view of phase gradient array.

The third region (Region III) is a one-dimensional (1D) phase-gradient particle chain, which acts as an output antenna for SPPs. As shown in Fig. 4, the particle unit consists of a metallic U-shaped structure and a grounded metallic sheet that are separated by a dielectric layer. From the antenna principle, periodically discontinuous particle loading in a slow-wave open structure will produce spatially harmonic wave. The mutual interaction of particles is responsible for mode-coupling resonances, thus affecting the operating frequency, radiation properties, and scanning angles. Our analysis starts from a simple 1D phase-gradient U-shaped particle array in Regine III, which can be seen as a local wave-vector modulator provided by the phase-gradient metasurface, leading to a transformation from SPPs to leaky waves.

The interaction mechanism for SPP radiation field can be obtained by a rigorous dispersion analysis. In Fig. 2, 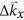 represents the difference of wave vectors between vacuum and corrugated metallic strip at the same frequency. Therefore, if we want to convert the SPP mode to radiation mode, we need to reduce the wave vector of SPPs. According to the generalized Snell’s law^31^, the horizontal wave vectors on the connection interface of two regions must satisfy the following relation


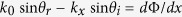


where *d*Φ is the phase discontinuity (i.e., phase difference 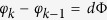) at a local point brought by the particle cell, *k*_*x*_(*k*_*spp*_) is the wave number of the corrugated metallic strip, while *θ*_*i*_ and *θ*_*r*_ are the incidence and deflection angles. When SPPs propagate along the gradient particle array with periodic configuration (see Fig. 1(b)), the wave number will be changed from *k*_*spp*_ to *k*_0_ with the deflection angle *θ*_*r*_. Changing the particle unit dimension, the phase of the deflected wave can be adjusted to an arbitrary value (within 2π range,see Fig. 4(b)).

From above discussions, by adjusting the wave vector, it is possible to control the angle of deflection. For broadside radiation (*θ*_r_ = 0) with horizontal incidence (*θ*_*i*_ = 90°), we have 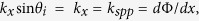 where *k*_*spp*_ is the SPP wave number. Therefore, by varying the geometric parameters of particle units, the gradient-phase array can function as a beam steering device, reaching both forward and backward quadrant scanning. Considering that the unit cells of 1D gradient-phase arrays are slowly modulated to obtain gradient-phase, the structure is considered as quasi-periodic. We set periodic boundary conditions for the unit cell to calculate the dispersion relation by using the Eigenmode Solver in the commercial software, CST Microwave Studio. Therefore, once the unit size is determined by phase difference simulation, we can calculate dispersion relation and surface impedance^34^,^35^. Choosing working frequency as *f* = 14 GHz, we have *k*_*x*_ = 1.326 *k*_0_ from the Fig. 2. Then we design the gradient-phase array with 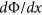 = −1.326 *k*_0_. Each period in Fig. 1(b) consists of six U-shaped particles, as shown in Fig. 4(a), in which the width of each particle is 2.7 *mm*. When the arm length of the U-shaped particle gradually decreases from 3.62 *mm* to 2.3 *mm*, the wave number of gradient-phase array will have an efficient transition from the SPP wave to free-space radiation, as illustrated in Fig. 4(c). Meanwhile, in order to satisfy the impedance matching, we also add an additional pair of the U-shaped particles between the corrugated metallic strips and 1D gradient-phase arrays, as demonstrated in Fig. 1(b).

In the above design, both of wave vectors and field modes are matched along the SPP-propagation direction, or the direction of periodicity. Increasing the number of repeated cycles, the proposed structure will enlarge the effective radiation aperture to achieve a higher directivity. Here, the number of repeated cycles was obtained as 4 by optimization the corresponding structures. The periodicity adds a degree of freedom in the design of radiation property. In observing the near electric fields on a plane perpendicular to the U-shaped particle array, as seen in Fig. 3(d), we notice that most of the EM fields spread out the particle units, and the energy coupling ratio in each single branch is nearly 50%.

**Figure 5:**
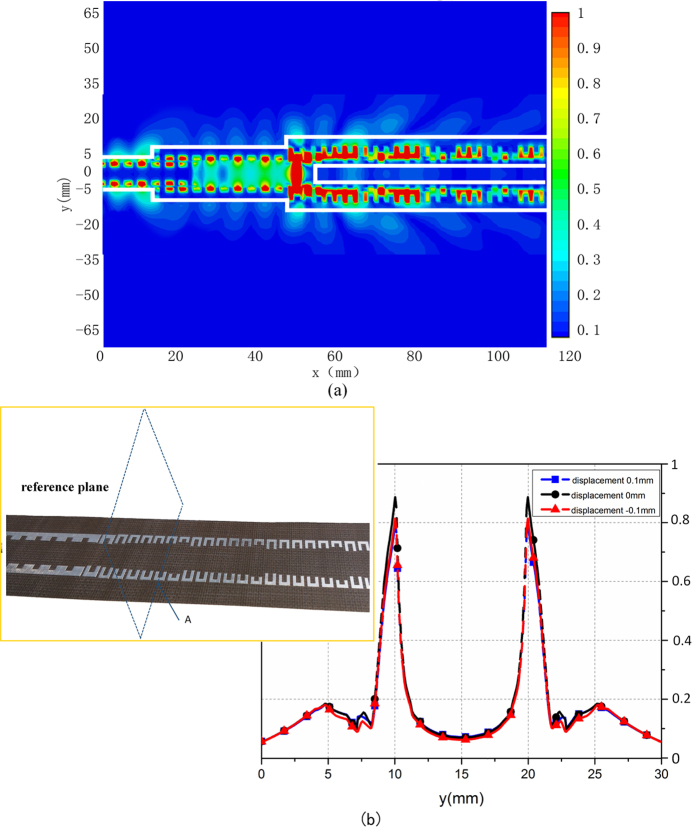
(**a**) The simulated near-electric-field distribution of the entire feeding and radiation structures on the *x–y* plane. (**b**) The intensity profiles of near electric fields along the observation lines at three positions (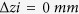, 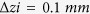 and 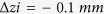).

In order to get more insights on the nature of the hybridized SPP and spatial modes occurring on the two kinds of SPP transmission lines and gradient U-shaped particle array, the electric field intensities of the whole system in the vicinity of the metallic surfaces are further analyzed and investigated. We calculate the *E*_*x*_ field profile at 14 GHz in the *x-y* plane that is 0.5 mm above the top metallic surface of the whole structure, as shown in Fig. 5(a). We note that the fields are confined around the subwavelength grooves along such two kinds of corrugated strips, have strong coupling between the two single-side corrugated strips in Region II, and are leaked to the space in Region III. For quantitative descriptions, we have extracted the *E*_*x*_ field intensity profiles along the vertical cuts (see blue dashed loop in Fig. 5(a)) on the *x-y* plane, as demonstrated in Fig. 5(b). When the reference plane (the blue dashed loop) is slightly shifted upward (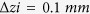) and downward (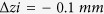), the curves of field profiles are nearly unchanged. We further find that the electric fields are symmetrically distributed on both sides of the corrugated structure, implying excellent performance of the plamsmonic power splitter.

**Figure 6:**
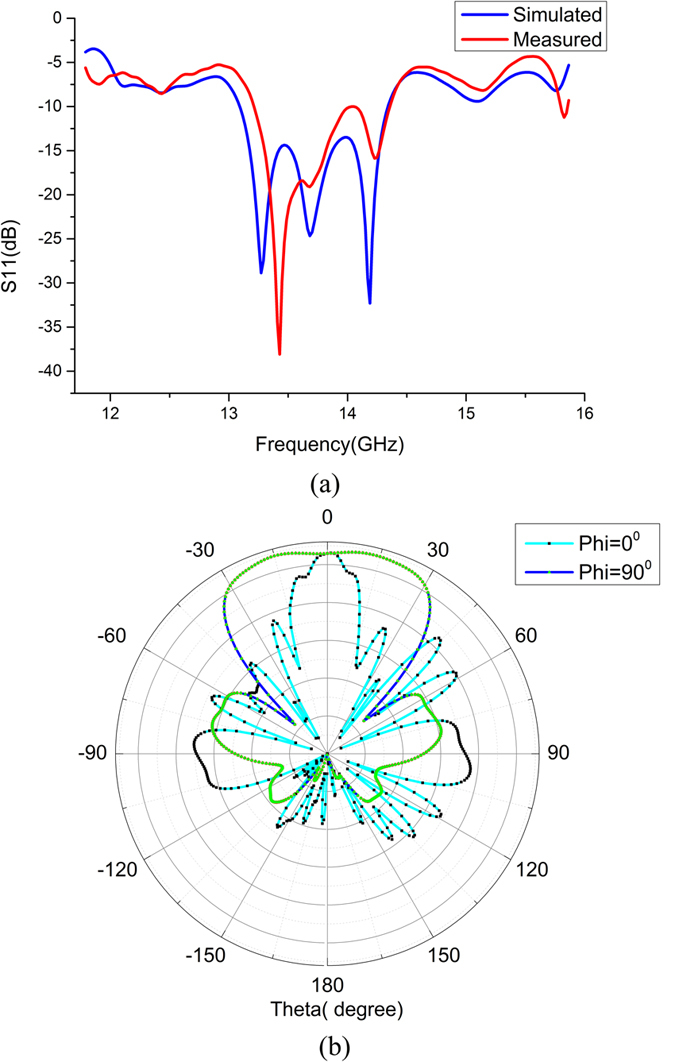
(**a**) The simulated and measured reflection coefficients (S_11_) at the CPW input port for the entire feeding and radiation structure. (**b**) The measured broadside far-field radiation patterns for Phi = 0° and Phi = 90° at the frequency of 14 GHz.

The validity of the proposed SPP radiation array and feeding network has been verified by both numerical simulations and experimental results. In experiment setup, we use a vector network analyzer (Agilent N5230C) to measure the reflection coefficients (S_11_) at the CPW input port. The comparison between the simulation and measurement results are presented in Fig. 6(a), in which the measured values have good agreements with simulations. From the measured reflection coefficients, we observe a high-efficiency SPP transmission along the waveguides and conversion from SPP to spatial modes, since S11 is lower than −10 dB in a relatively wide band. In fact, most of the EM energy is radiated to the space by the two parallel arrays, forming broadside far-field radiation patterns, as illustrated in Fig. 6(b).

## Discussion

In summary, we have proposed a splitter feeding network and gradient-phase radiation particles for array radiations of spoof SPPs in the broadside direction based on ultrathin metallic structures. We investigated in details the influences of SPP groove parameters on the mutual coupling, and the physical mechanism of U-shaped particle chain as array antenna. To verify the performance of the 1 × 2 feeding network, we have implemented experiments on the fabricated prototypes, and the measured performance clearly validated the functionality of the designs. The experiments showed that two channels can be efficiently driven by a single SPP transmission line. The proposed plasmonic feeding network is helpful to realize other planar SPP devices and high-gain antennas. By incorporating actively tunable, switching, and/or modulation capabilities into the plasmonic feeding network, the proposed method can be extended to SPP beam scanning and beam shaping.

## Methods

Numerical simulations are performed by the commercial software, CST Microwave Studio. The experimental structure is fabricated using a 1 mm thin dielectric film with dielectric constant 3.5 and tangent loss 0.005, respectively. The thickness of metal (copper) film is 0.018 mm. We use Agilent Vector Network Analyzer to measure the S parameters (i.e., the reflection coefficients S_11_ of the fabricated sample.) The near electric-filed distributions are measured by a home-made near-field scanning system, in which the probe is set as 0.5 mm above the fabricated sample.

## Additional Information

**How to cite this article**: Xu, J. J. *et al.* Compact Feeding Network for Array Radiations of Spoof Surface Plasmon Polaritons. *Sci. Rep.* **6**, 22692; doi: 10.1038/srep22692 (2016).
